# Choosing the optimal spectroscopic toolkit to understand protein function

**DOI:** 10.1042/BSR20160378

**Published:** 2017-06-07

**Authors:** Michael A. Hough

**Affiliations:** School of Biological Sciences, University of Essex, Wivenhoe Park, Colchester, Essex, CO7 6SR, U.K.

**Keywords:** Combined methods, EPR spectroscopy, NMR spectroscopy, Protein spectroscopy, structural biology, Time-resolved spectroscopy

## Abstract

Spectroscopy was one of the earliest methods used to study the properties and reactions of proteins, and remains one of the most powerful and widely used approaches to this day. A sometimes bewildering range of spectroscopies is now available, applicable to different sample states, timescales and indeed biological questions. This editorial describes some of the most relevant spectroscopic methods together with a selection of illustrative examples.

A wide array of spectroscopic tools are now available to characterize the properties of proteins in solution and crystalline states. A considerable challenge for a researcher with limited resources and time is to select the most appropriate set of spectroscopies to address a particular biological or biochemical question. Important factors to consider are the state of the protein (typically solution or crystal), the time domain to be explored, the presence or absence of a spectroscopically accessible chromophore and the relevance of a particular *in vitro* spectroscopy to protein function *in vivo*. Different spectroscopies provide information at the global, local or atomic level and in general, for maximum biological insight, it is essential that a suitable combination of spectroscopies are applied to answer the most relevant questions. Often, spectroscopic data of proteins are interpreted in combination with structural biology or *in vivo* studies.

Certain spectroscopies can probe secondary structure and folding in essentially any protein. Typically, Fourier transform IR (FTIR) [1] or circular dichroism (CD) [2,3] approaches are used, providing an excellent dynamic monitor of protein folding/unfolding states as well as, in a static experiment, yielding information on the secondary structure composition of a protein (α-helical, β-sheet and loop). Such techniques have been successfully applied to extract midpoint melting transition temperatures and energies of unfolding as well as monitoring chemical denaturation of protein samples.

Where chromophore cofactors and/or metal centres are present, a rich array of spectroscopies may be brought to bear. The most common chromophores found in proteins are haem, flavins, NAD/NDP/NAP or metal ions [4,5]. The most commonly applied approach is UV-Visible absorption spectroscopy (UV-Vis), a method that is highly sensitive to chromophore electronic states related to ligation, redox state and pH. The wavelength range used in UV-Vis is typically 250–700 nm, which accesses many common protein chromophore electronic transitions. Extending spectroscopy into the IR range allows for vibrational information to be gained [6], although experiments are complicated by the strong absorbance of IR light by water.

Raman spectroscopy also reports the vibrational properties of proteins [7]. Simple Raman spectroscopy with an excitation laser that is not tuned to a particular chomophore reports on the vibrational properties of the peptide backbone and side chains, leading to a very large number of bands being observed for macromolecules with assignment of individual bands to particular amino acids being challenging. A particularly useful application for chromophore-containing proteins is resonance Raman spectroscopy, where the excitation laser is tuned to an electronic absorption maximum of the chromophore. This leads to strong amplification of the chromophore-related peaks and has been applied with particular success to study haem proteins, with excitation into the Soret absorption band. Further dynamical information may be obtained using 2D IR approaches [8] that can yield information on protein transient structures and dynamics. This spectroscopy measures the effect of an excitation on other molecular vibrations at selected time points and is most typically applied in proteins to amide vibrational modes.

Deeper electron energy levels may be explored using X-ray absorption (XAS) spectroscopy [9], which can provide direct metrical information for metal (or S) co-ordination shells and geometry. Notably, this elemental selectivity allows different metal centres within a protein to be separately studied. XAS is informative for a radius of ∼5 Å from the absorbing atom and has the great advantage that a spectrum may be measured for any element for which the appropriate X-ray energy is experimentally accessible [10]. Recent work has combined electrochemistry with XAS [11] to access multiple redox states with future applicability to redox proteins. Examples of XAS to proteins include characterization of the structure and different redox states of the Mn cluster in photosystem II [12] and extensive contributions to understand the nature of the iron-molybdenum cofactor in nitrogenase (reviewed in [13]).

EPR spectroscopy is highly applicable to paramagnetic proteins, providing a powerful probe of the spin properties of, and environment around, metal or radical sites that are relevant to functional mechanisms [14]. An interesting example is the identification of which amino acid is the site of a tyrosyl radical using density functional theory simulations based on a crystal structure to simulate the rotational angle of the side chain and its effect on the radical signal [15].

NMR spectroscopy is a very powerful and versatile spectroscopy with many applications to proteins. NMR has been applied, for example, to the detection of ligand–protein binding [16], to study protein structural dynamics [17,18], protein folding [19], to predict secondary structures [20] and to characterize protein–protein interactions [21,22]. NMR can provide atomic-level information and can be used to determine the 3D structures of smaller proteins and other macromolecules [23,24].

The speed with which spectra may be collected is a relevant factor and should be considered in comparison with the rates of enzyme reactions and other processes. Time-resolved approaches are particularly applicable to the analysis of enzyme kinetics, for example when coupled to stopped-flow rapid mixing apparatus, using freeze-quench methods or more straightforwardly to slow time courses using continuous time course measurement of spectra. Stopped-flow methods are also applicable to time-resolved studies of protein folding [25], with the mixed sample monitored by CD or fluorescence spectroscopies. Laser flash photolysis as a means of reaction initiation allows enzyme reactions and other processes to be followed at extremely rapid timescales. At the shortest timescales (femtosecond and increasingly, attosecond), ultrafast processes such as geminate recombination of gas ligands to haem proteins may be followed using laser-induced bond breakage or reaction initiation [26]. Most commonly employed as a pump-probe method using UV-Vis [27,28], other rapid spectroscopies have also been developed. Recent examples include the use of time-resolved IR (TRIR) spectroscopy to study steps in the photocycle of channel rhodopsin over a broad timescale (reviewed in [29]) and the application of time-resolved resonance Raman (TRRR) spectroscopy to characterize ultrafast (ps) protein dynamics associated with ligand photolysis in haemoglobin [30].

Where 3D structures of proteins are determined by X-ray crystallography, relatively recent developments have allowed spectroscopic data to be obtained from the same X-ray exposed region of the crystal from which the structure is determined. This allows comparison of solution and crystalline protein and allows the effects of X-ray induced changes to the sample to be monitored. This approach is highly synergistic for redox-sensitive chromophores such as transition metals or flavins [31,32]. The most commonly applied spectroscopy at beamlines is UV-Visible absorption, with several instruments also allowing Raman (resonance and non-resonance) and fluorescence data collection. Single crystal Raman spectroscopy in concert with X-ray crystallography has been used to provide direct evidence for a peroxide intermediate in uricase, a cofactor-free oxidase [33]. A useful example of single crystal UV-Vis was its use to identify the deprotonation of a bilin chromophore in a bacterial phytochrome in response to extremely low doses of X-rays [34]. The present study highlighted the importance of spectroscopic validation of the state of chromophores in crystal structures.

X-ray absorption methods may also be applied to protein crystals, either alone or in combination with X-ray diffraction from the same sample [35]. This approach is widely used to identify optimal X-ray wavelengths for structure determination by anomalous dispersion (SAD or MAD) methods but with suitable instrumentation can allow high quality X-ray absorption near edge structure (XANES) data to yield insight into the redox state and local environment of the absorbing atom. XAS-derived parameters may be used to restrain crystallographic refinement in the MXAN [36] approach, while crystal structures can provide models for interpretation of EXAFS data in the 3D-EXAFS method [37,38]. An excellent recent example of combined solution of EXAFS and XANES spectroscopies used to address a key biological problem was a study of the pathological Q212P mutation upon the octarepeat Cu-binding region of the human prion protein [39]. These combined X-ray absorption methods indicated substantial changes to Cu co-ordination in comparison with the native protein, suggesting a loss of redox control in the mutant as a pathogenic mechanism.

Similar approaches applied to serial femtosecond crystallography experiments at X-ray free electron laser sources (XFELs) are useful to measure data from microcrystals that are destroyed during the very brief time of data collection. X-ray emission spectroscopy (XES) [40] is able to provide spectroscopic information about metal redox state and ligand environment precisely at the point that the sample and X-ray beam interact. A parallel development is measurement of X-ray absorption data in a similar manner. A recent example was the observation of haem doming in myoglobin using time-resolved XAS [41].

In summary, spectroscopic tools provide a powerful means to understand the diverse functions of proteins, but careful selection of spectroscopy, time-regime and sample conditions are essential to make the best use of these.

## Abbreviations

MAD: multi-wavelength anomalous dispersion
NDP: Dihydro-nicotinamide-adenine dinucleotide phosphate
NAP: Nicotinamide-adenine dinucleotide phosphate
S: sulfur
SAD: single wavelength anomalous dispersion
UV-Vis: UV-Visible absorption spectroscopy
XANES: X-ray absorption near edge structure
XAS: X-ray absorption spectroscopy
